# Amelioration of Cognitive Deficit by Embelin in a Scopolamine-Induced Alzheimer’s Disease-Like Condition in a Rat Model

**DOI:** 10.3389/fphar.2018.00665

**Published:** 2018-06-25

**Authors:** Saatheeyavaane Bhuvanendran, Yatinesh Kumari, Iekhsan Othman, Mohd Farooq Shaikh

**Affiliations:** Neuropharmacology Research Laboratory, Jeffrey Cheah School of Medicine and Health Sciences, Monash University Malaysia, Bandar Sunway, Malaysia

**Keywords:** embelin, Alzheimer’s disease, cognition, neuroprotective, anti-amnesic effect

## Abstract

Embelin (2,5-dihydroxy-3-undecyl-1,4-benzoquinone) is one of the active components (2.3%) found in *Embelia ribes* Burm fruits. As determined via *in vitro* AChE inhibition assay, embelin can inhibit the acetylcholinesterase enzyme. Therefore, embelin can be utilized as a therapeutic compound, after further screening has been conducted for its use in the treatment of Alzheimer’s disease (AD). In this study, the nootropic and anti-amnesic effects of embelin on scopolamine-induced amnesia in rats were evaluated. Rats were treated once daily with embelin (0.3 mg/kg, 0.6 mg/kg, 1.2 mg/kg) and donepezil (1 mg/kg) intraperitoneally (i.p.) for 17 days. During the final 9 days of treatment, a daily injection of scopolamine (1 mg/kg) was administered to induce cognitive deficits. Besides that, behavioral analysis was carried out to assess the rats’ learning and memory functions. Meanwhile, hippocampal tissues were extracted for gene expression, neurotransmitter, and immunocytochemistry studies. Embelin was found to significantly improve the recognition index and memory retention in the novel object recognition (NOR) and elevated plus maze (EPM) tests, respectively. Furthermore, embelin at certain doses (0.3 mg/kg, 0.6 mg/kg, and 1.2 mg/kg) significantly exhibited a memory-enhancing effect in the absence of scopolamine, besides improving the recognition index when challenged with chronic scopolamine treatment. Moreover, in the EPM test, embelin treated rats (0.6 mg/kg) showed an increase in inflection ratio in nootropic activity. However, the increase was not significant in chronic scopolamine model. In addition, embelin contributed toward the elevated expression of BDNF, CREB1, and scavengers enzymes (SOD1 and CAT) mRNA levels. Next, pretreatment of rats with embelin mitigated scopolamine-induced neurochemical and histological changes in a manner comparable to donepezil. These research findings suggest that embelin is a nootropic compound, which also possesses an anti-amnesic ability that is displayed against scopolamine-induced memory impairment in rats. Hence, embelin could be a promising compound to treat AD.

## Introduction

Alzheimer’s disease (AD) is known as the leading cause of dementia amongst people aged 65 and older (Ghumatkar et al., 2015). This age-related disease affects millions of individuals, and it is estimated that by 2050, 1 in 85 people worldwide will be suffering from AD (Brookmeyer et al., 2007). According to Tanzi and Bertram (2005), AD is a progressive and chronic neurodegenerative disorder which displays global cognitive decline involving memory, orientation, judgment, and reasoning. The key features of AD’s pathogenesis are the gradual amassing of the protein fragment beta-amyloid (plaques) and twisted fibers of the protein tau (tangles), outside and inside neurons in the brain, respectively (Alzheimer’s Association, 2017). Beta-amyloid plaques function as a neurotoxin by intervening in neuron-to-neuron communication at synapses. On the other hand, tau tangles prevent the passage of essential molecules and nutrients inside neurons, which causes axonal transport dysfunction and neuronal loss (Ali et al., 2015; Alzheimer’s Association, 2017).

Apart from that, memory impairment is associated with cholinergic system dysfunction, which involves cholinergic neurons, neurotransmitters, and their receptors (Bartus et al., 1982; Lee et al., 2015). Cholinergic system dysfunction results from a loss of cholinergic neurons in the basal forebrain and hippocampus, which diminishes cognitive capability (Bartus et al., 1982; Lee et al., 2015). In healthy individuals, activation of the central cholinergic system enhances hippocampal neurogenesis through the cAMP response element-binding protein/brain-derived neurotrophic factor (CREB/BDNF) pathway (Lee et al., 2015). At present, one of the treatments for AD is a dispensation of acetylcholinesterase (AChE) inhibitors like tacrine or donepezil that increase the availability of acetylcholine at cholinergic synapses (Pandareesh et al., 2016). Moreover, oxidative stress plays an important role in AD, with some studies suggesting that beta-amyloid toxicity is linked to an increment in reactive oxygen species (ROS), including H_2_O_2_ (Butterfield and Lauderback, 2002), and lipid peroxidation in neuronal cultures (Yatin et al., 1999). High oxidative stress can cause memory deficits via impairment of hippocampal synaptic plasticity (Serrano and Klann, 2004) and oxidative damage in neurodegenerative diseases (Ding et al., 2007).

Current pharmacological options for AD, only have a partial effect and poor control over the disease-causing neurons linked with Alzheimer’s symptoms and lethal complications (Alzheimer’s Association, 2017). As such, the available drugs in the market mainly focus on the improving memory by inhibiting the AChE enzyme (Ghumatkar et al., 2015). However, AD is not a result of a single factor like AChE, but rather is a multifactorial condition and this needs to be considered when designing a drug. Other factors such as oxidative stress and synaptic dysfunction play a significant role in the cognitive deficits in AD. Natural products could be a source of neuroprotective drugs as they can maintain normal cellular interaction in the brain and reduce the loss of neuronal functions in pathological circumstances (Hritcu et al., 2014). Presently, many AD research groups have already explored the potential of using natural products as neuroprotective agents.

One such potential natural product is embelin (2,5-dihydroxy-3-undecyl-1,4-benzoquinone), which is the main active constituent in the fruits of *Embelia ribes* Burm (Family: Myrsinaceae), commonly known as “False Black Pepper” (Kundap et al., 2017a). The bright orange fruits of *E. ribes* have been utilized in traditional medicinal practice for treating central nervous system (CNS) disorders such as mental disorders and as a brain tonic (Poojari, 2014). Moreover, embelin has displayed anti-inflammatory, antioxidant, analgesic, antifertility, antitumor, wound healing, hepatoprotective, and antibacterial activities (Mahendran et al., 2011). Additionally, it has been reported that embelin is neuroprotective and possesses anticonvulsant ability when tested using animal models (Mahendran et al., 2011).

Embelin possesses all the features of a compound that can traverse the blood-brain barrier (BBB) and prompt a reaction in the CNS (Pathan et al., 2009; Kundap et al., 2017a). Even though embelin has various uses, there have been no studies of its neuropharmacological activities against AD-like conditions. Thus, in the present study, the anti-amnesic potential of embelin on memory deficits in a rat model of cognitive impairment caused by scopolamine was examined.

## Materials and Methods

### Animal Care

In-house bred Sprague Dawley rats weighing between 180–200 g and between 6–8 weeks old were housed in the animal facility of the Jeffrey Cheah School of Medicine and Health Sciences, Monash University Malaysia. The rats were kept in cages and maintained under standard husbandry conditions (12:12 h light/dark cycle, controlled room temperature (23 ± 2°C), stress-free, *ad libitum* water, standard diets, and sanitary conditions). Before commencing the experiment, the rats were allowed to acclimatize for a period of 1 week to reduce stress. The Monash Animal Research Platform (MARP) Animal Ethics Committee in Australia approved all the animal experimentations conducted in this study.

### Experimental Design

#### Drug Treatment

Embelin (98%) batch number (Yucca/EM/2015/01/01) was purchased from Yucca Enterprises, Mumbai, India. The range of doses for embelin was determined based on pre-screening results. Embelin was solubilized in DMSO and then dissolved in saline. Donepezil and scopolamine were prepared in saline. Normal control rats were administered saline throughout the experiment. The treatments were given intraperitoneally (i.p) at a volume corresponding to 0.1 ml/100 g of body weight.

All experiments were performed in a balanced design (9 animals/group) to avoid being influenced by order and time. The behavioral studies were divided into two categories namely the nootropic and scopolamine models.

#### Nootropic Model

(i) **Group 1**: Control (Saline) (*n* = 9);
(ii) **Group 2**: Positive control (donepezil (DPZ) 1 mg/kg);
(iii) **Group 3**: Low dose of embelin (EMB) 0.3 mg/kg;
(iv) **Group 4**: Medium dose of EMB 0.6 mg/kg;
(v) **Group 5**: High dose of EMB 1.2 mg/kg

**FIGURE 1:**
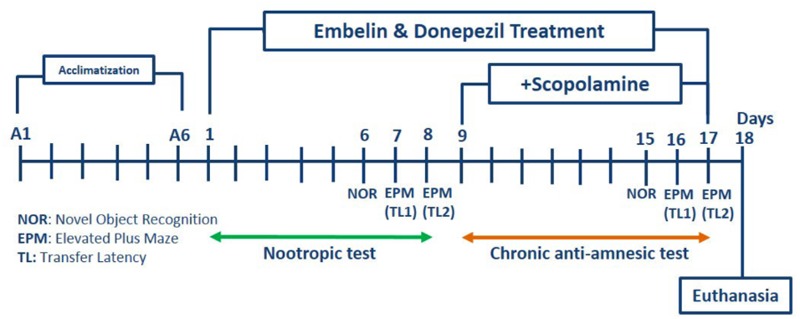
Schematic representation of the experimental procedure.

For nootropic activity, all the groups received pretreatment via the intraperitoneal route, for 8 days. All these rats were subjected to a battery of behavioral tests from day six onward until day eight for NOR and EPM (**Figure 1**).

#### Scopolamine Model

(i) **Group 1**: Control (Saline) (*n* = 9);
(ii) **Group 2**: Negative control [scopolamine (SCP) 1 mg/kg] (*n* = 9);
(iii) **Group 3**: Positive control [donepezil (DPZ) 1 mg/kg] + (SCP 1 mg/kg) (*n* = 9);
(iv) **Group 4**: Low dose of embelin (EMB) 0.3 mg/kg + (SCP 1 mg/kg) (*n* = 9);
(v) **Group 5**: Medium dose of EMB 0.6 mg/kg + (SCP 1 mg/kg) (*n* = 9);
(vi) **Group 6**: High dose of EMB 1.2 mg/kg + (SCP 1 mg/kg) (*n* = 9)

For scopolamine, amnesia was induced in all the groups except the control group by daily intraperitoneal injections of scopolamine (1 mg/kg) for 9 days after embelin pre-treatment (day nine to day 17). Half an hour after scopolamine administration, NOR was conducted on day 15, and EPM was carried out on day 16 and 17 of the study. At the end of the experiment, the rats were sacrificed, and their brains were isolated for further biochemical and immunohistochemistry analysis.

### Novel Object Recognition (NOR)

For the object recognition task, an open field box (40 × 40 × 20 cm) composed of black acrylic material was utilized as the experimental apparatus. This method is similar to that used by Ennaceur and Delacour (1988), with minor modifications. Besides that, behavioral testing was carried out between 9:00 am and 6:00 pm under red light illumination. The scrutinized objects were two similar transparent culture flasks containing water and a Lego toy of similar height as that of the flask (new object). Both objects types presented during the test session varied in texture, color, and size. This assessment has three phases: (i) habituation; (ii) training, and (iii) test. On the first day, each rat was allowed to become familiarized with the open field box without the presence of an object for about 10 min. On the second day, each rat was placed in the open field for 5 min and allowed to freely explore the two identical objects (transparent cultured flask with water). After an interval of 90 min post-training session, one of the old objects used was substituted with a new object and the rats were subjected to a 2 min test run. The time spent with each object was recorded and evaluated using SMART software version 3.0. The open field box was cleaned with 70% ethanol between runs to minimize scent trails. The recognition index was calculated using the formula [TB/(TA + TB)^∗^100] where TA and TB are time spent exploring familiar object A and novel object B respectively (Batool et al., 2016). Exploration of an object was noted when a rat sniffed or touched the object with its nose and/or forepaws.

### Elevated Plus Maze (EPM)

The EPM device was comprised of four arms sharing the same dimensions, i.e., two open arms (50 × 10 cm) that crossed over two closed arms with 40 cm high walls. These arms were connected using a central square (10 × 10 cm), thus giving the apparatus plus sign look. Furthermore, the EPM was elevated 50 cm above floor level. This technique is almost similar to one reported by Halder et al. (2011). The behavioral testing was conducted between 9:00 am and 6:00 pm under dim red light illumination. Assessment of memory via EPM was done in two sessions. During the training phase, each rat was placed at the end of an open arm and by using a stopwatch, transfer latency time (s), which is the time each rat took to enter (with all four paws) into either closed arm, was noted. The maze was cleaned with 70% ethanol between runs to minimize scent trails. To evaluate memory retention, a test phase was conducted 24 h (retention) after a training session. The cut-off time for each rat to explore the maze in both the phases (training and test) was 90 s. A drop in transfer latency time during test sessions was taken as an index of memory improvement.

### Tissue Processing

All the rats were sacrificed under ketamine and xylazine anesthesia 1 h after completing the behavioral test. In each group, five rat brains were fixed in 4% paraformaldehyde, and hippocampi of remaining four rats were used for real-time PCR and neurotransmitter analysis. One part of the hippocampus was used for isolation of RNA and another part of the hippocampus was homogenized on ice using methanol containing formic acid.

### Total RNA Extraction and Real-Time PCR

Total RNA was extracted from the rat brain’s hippocampal region and was similar to the method used by Kundap et al. (2017b), with some minor modifications. One part of the hippocampus tissue was momentarily homogenized in Trizole solution. The mixture was extracted using chloroform and centrifuged at 13,500 rpm at 4°C. Then, the aqueous phase was precipitated with isopropanol and followed by centrifugation at 13,500 rpm at 4°C. The volume of isopropanol added was same as the volume of the supernatant from the aqueous phase. After that, the alcohol was removed. The pellet on the other hand, was rinsed twice with 70% ethanol and resuspended in 20 μL of RNase free water. RNA concentration was ascertained via absorbance at 260 nm using a Nanodrop machine. The total RNA (500 ng) was then reverse transcribed to synthesize cDNA using a QuantiTect^®^ Reverse Transcription Kit, according to the manufacturer’s protocol. Next, the mRNA expression of genes encoding cAMP response element-binding protein (CREB1), brain-derived neurotrophic factor (BDNF), superoxide dismutase 1 (SOD1), catalase (CAT), and IMPDH2 in the hippocampus, was measured by real-time PCR using the StepOne Real-Time PCR system. Subsequently, cDNA from the reverse transcription reaction was subjected to real-time PCR using a QuantiNova^TM^ SYBR^®^ Green PCR kit according to manufacturer’s protocol. A comparative threshold (C_T_) cycle method was applied to normalize cDNA content of samples, which involves of normalization of a number of target gene copies against the endogenous reference gene, IMPDH2.

### Neurotransmitter Analysis Using Liquid Chromatography-Tandem Mass Spectrometry (LC-MS/MS)

The brain levels of neurotransmitters like dopamine (DA), glutamate (Glu), norepinephrine (NE), and acetylcholine (ACh) were estimated using LC-MS/MS in a similar manner to that used by Kundap et al. (2017b), with some modifications. For all these standard neurotransmitters, stock solutions of 1 mg/ml were prepared in methanol (0.1% formic acid) and then stored at 4°C until use. Four calibration standards with the concentration ranges of 0.25–200.00, 250.00–20,000.00, 0.50–200.00, and 0.25–200.00 ng/mL were used for validation of DA, Glu, NE, and ACh respectively. In brief, hippocampal tissue was homogenized in ice-cold methanol containing formic acid. Then, the homogenate was vortex-mixed followed by centrifugation at 14,000 rpm for 10 min at 4°C. Finally, the supernatant was subjected to LC-MS/MS analysis, which was run on an Agilent 1290 Infinity UHPLC, coupled with an auto-sampler system comprising of Agilent 6410 Triple Quad LC/MS, ZORBAXEclipse plus C18 RRHD 2.1 × 150.0 mm and 1.8-micron (P/N959759-902) column (Agilent Technologies, Santa Clara, CA, United States). The mobile phase consisted of 0.1% formic acid in (i) water (Solvent A) and (ii) acetonitrile (Solvent B). It was used with a gradient elution: (i) 0–3 min, 50% B; (ii) 3–6 min, 95% B; (iii) 6–7 min, 95% B at a flow rate of 0.1 mL/min.

### Immunohistochemical Stain Analysis

Immunohistochemical stain analysis was conducted via assessment of neurogenesis using Doublecortin (DCX) and lipid peroxidation with 4-hydroxy-2-nonenal (4HNE) staining in the hippocampus. Five brain samples from each group were immersed in 4% paraformaldehyde overnight. The samples were methodically cryoprotected in 10, 20, and 30% sucrose for 24 h. Next, the brains were embedded in 15% polyvinylpyrrolidone (PVP), frozen using dry ice, and cut into 40 μm frozen coronal sections using a Leica CM3050 cryostat. All sections were then stored in an anti-freeze buffer. Endogenous quenching using 1% H_2_O_2_ in methanol for 30 min was performed on the free-floating sections. After washing with phosphate buffered saline (PBS), the tissues were treated with blocking buffer (1.0% bovine serum albumin in PBS and 0.3% Triton X-100) for 1 h, followed by incubation with primary DCX (1:500, Abcam) and 4HNE (1:250, Abcam) antibodies overnight at 4°C. The tissues were then incubated with a biotinylated goat anti-rabbit secondary antibody (Abcam) for 2 h after being washed with PBS. Subsequently, the tissues were exposed for 2 h to an avidin-biotin-peroxidase complex (Vectastain ABC kit, Vector). Peroxidase activity was visualized using a stable diaminobenzidine solution (DAB, Sigma). All immunoreactions were monitored via a microscope (BX41, Olympus) and using the DigiAcquis 2.0 software, results were calculated.

### Statistical Analysis

All findings were expressed as mean ± standard error of the mean (SEM). These data were analyzed using one-way analysis of variance (ANOVA) followed by Dunnett’s tests. The *P*-values of ^∗^*P* < 0.05, ^∗∗^*P* < 0.01, and ^∗∗∗^*P* < 0.001 were considered as statistically significant. All the experimental groups were compared with the SCP 1 mg/kg group.

## Results

### Nootropic effect of Embelin

**FIGURE 2:**
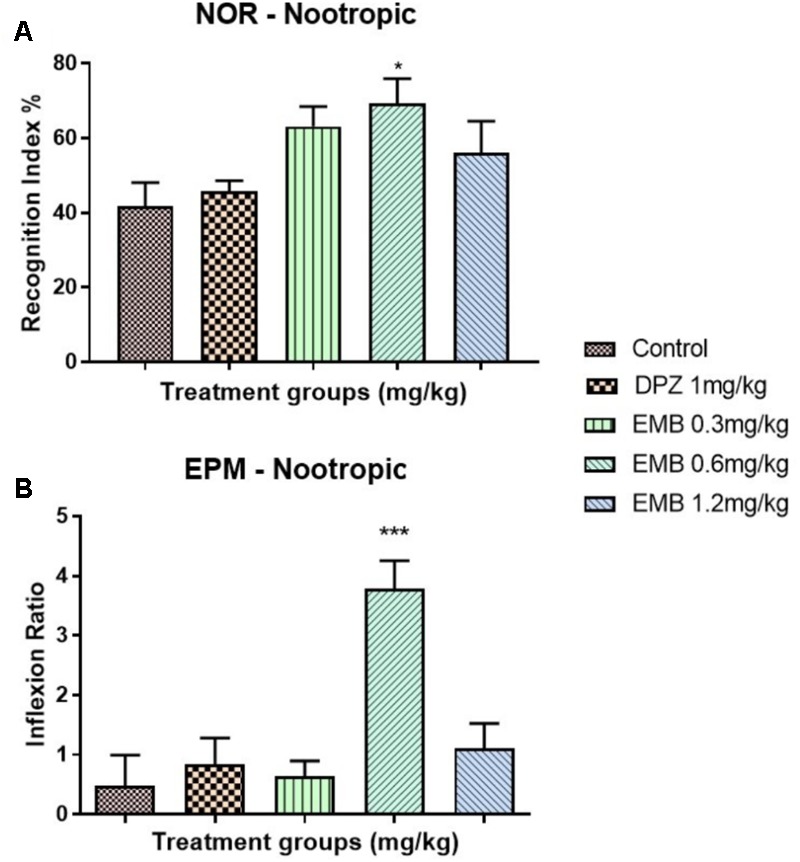
Behavioral analysis for novel object recognition (NOR) and elevated plus maze (EPM). **(A)** represent the graph plot for the recognition indices in NOR for the nootropic model **(B)** represents the graph plot for the inflection ratios in EPM for the nootropic model. The behavioral analysis for **(A,B)** were compared to control group. Data are expressed as Mean ± SEM, *n* = 9 and statistical analysis by one-way ANOVA followed by Dunnett test ^∗^*P* < 0.05, ^∗∗^*P* < 0.01, and ^∗∗∗^*P* < 0.001.

Findings obtained from the NOR test for embelin nootropic activity are illustrated in **Figure 2A**. The effect of different embelin doses on memory function were assessed following 7 days of pretreatment. The results were expressed as recognition index (%) for the novel object. Based on the outcomes, the pretreated groups of embelin showed an increase in recognition index for novel object compared with the control group and donepezil groups. Only 0.6 mg/kg of embelin showed statistically significant results with p value of <0.05. In EPM, the inflection ratio was significantly increased in 0.6 mg/kg embelin treated groups when compared with the control (**Figure 2B**). There was no significant difference in other treated groups.

### Anti-amnesic Effect of Embelin in Rats With Scopolamine-Induced Amnesia

**FIGURE 3:**
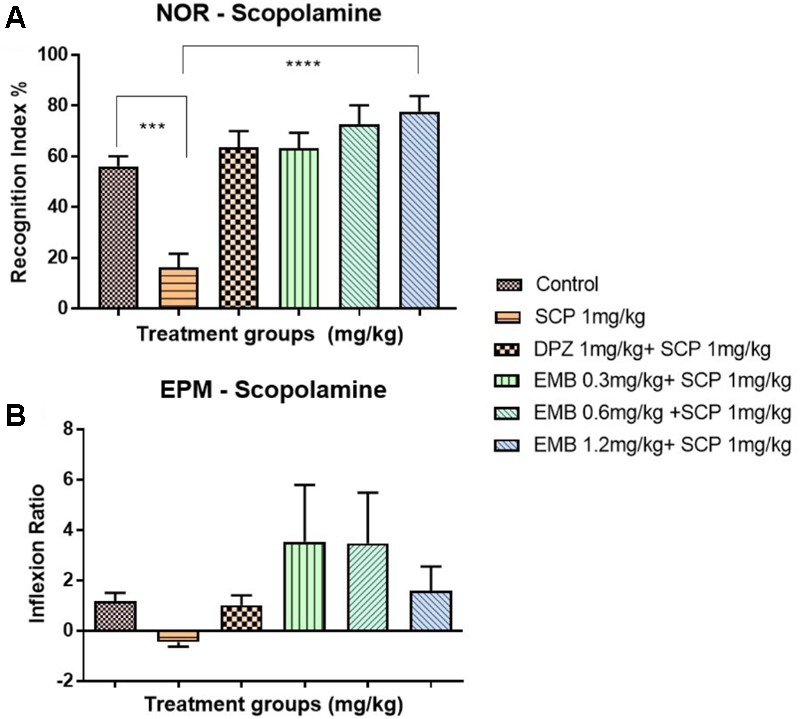
Behavioral analysis for NOR and EPM. **(A)** represents the graph plot for the recognition indices in NOR for the scopolamine model **(B)** represents the graph plot for inflection ratios in EPM for the scopolamine model. The behavioral analysis for **(A,B)** were compared to negative control (SCP 1mg/kg). Data are expressed as Mean ± SEM, *n* = 9 and statistical analysis by one-way ANOVA followed by Dunnett test ^∗^*P* < 0.05, ^∗∗^*P* < 0.01, ^∗∗∗^*P* < 0.001, and ^∗∗∗∗^*P* < 0.0001.

The NOR test showed a reduction in recognition index percentage for the negative group (SCP 1 mg/kg) in the chronic scopolamine model (**Figure 3A**). Moreover, the recognition index percentage for all the embelin treated groups were high and comparable with donepezil (1 mg/kg) group. A significant difference in the recognition index percentage was observed between all embelin treated and the negative group (*P* < 0.05). In EPM, inflection ratio analysis showed that there was an increase in retention memory in all embelin treated groups compared with the negative control group; however, it was statistically not significant (**Figure 3B**).

### Changes in mRNA Levels in the Hippocampus

**FIGURE 4:**
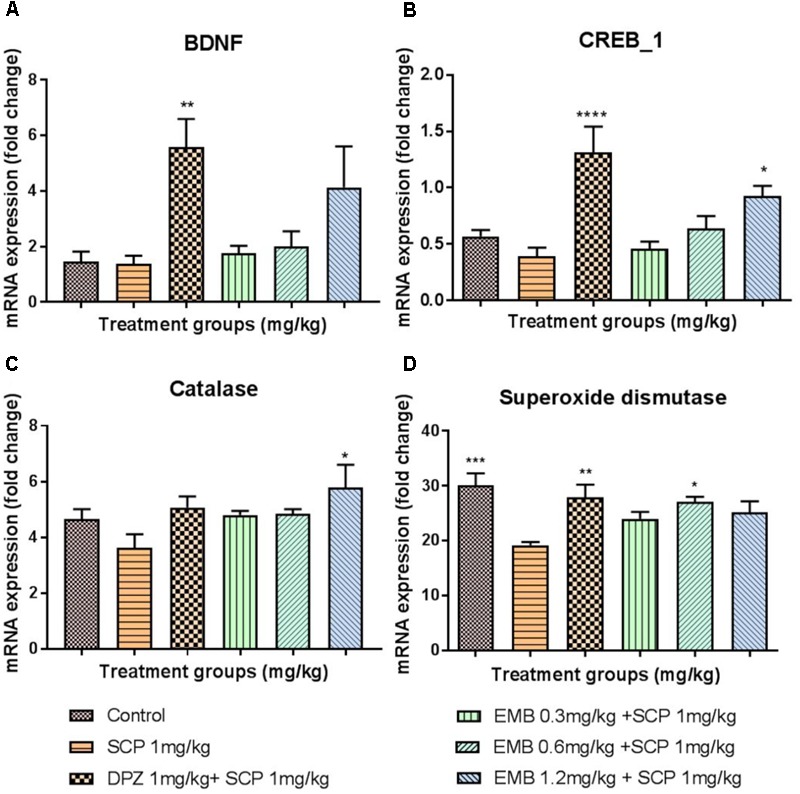
Gene expression in the rat hippocampi determined by real time-PCR. The genes included are **(A)** BDNF, **(B)** CREB1, **(C)** Catalase, and **(D)** Superoxide Dismutase. All changes in the expressions levels were compared to the negative control group (SCP 1 mg/kg). Data are expressed as Mean ± SEM, *n* = 4 and statistical analysis by one-way ANOVA followed by Dunnett test ^∗^*P* < 0.05, ^∗∗^*P* < 0.01, ^∗∗∗^*P* < 0.001, and ^∗∗∗∗^*P* < 0.0001.

BDNF mRNA levels were significantly down-regulated, approximately twofold in the hippocampus of the scopolamine group, compared with the positive control, *P* < 0.01. This down-regulation was ameliorated by embelin, in a dose-dependent manner, in comparison with the negative control, and a significant difference was revealed for the 1.2 mg/kg dose of embelin (**Figure 4A**). In addition, multiple exposures to scopolamine significantly down-regulated (twofold) the mRNA expression level of CREB1 in the negative control, compared with the positive control (*P* < 0.001). Embelin treatment increased CREB1 expression level in a dose-dependent manner, compared with the negative control, and it was significant for the 1.2 mg/kg embelin dose (**Figure 4B**). Furthermore, scopolamine depleted antioxidant mRNA in hippocampal tissues, including (CAT) (**Figure 4C**) and SOD1 gene expression (**Figure 4D**). The down-regulation of CAT mRNA was significantly ameliorated through embelin treatment compared to the negative control for the 1.2 mg/kg embelin group (*P* < 0.05). In SOD1, these changes were reversed by embelin pretreatment for all embelin treated groups, and the result was significant in the 0.6 mg/kg embelin group, with approximately a 1.5-fold change in comparison with the scopolamine treated group.

### Estimation of Neurotransmitters by LC-MS/MS

**FIGURE 5:**
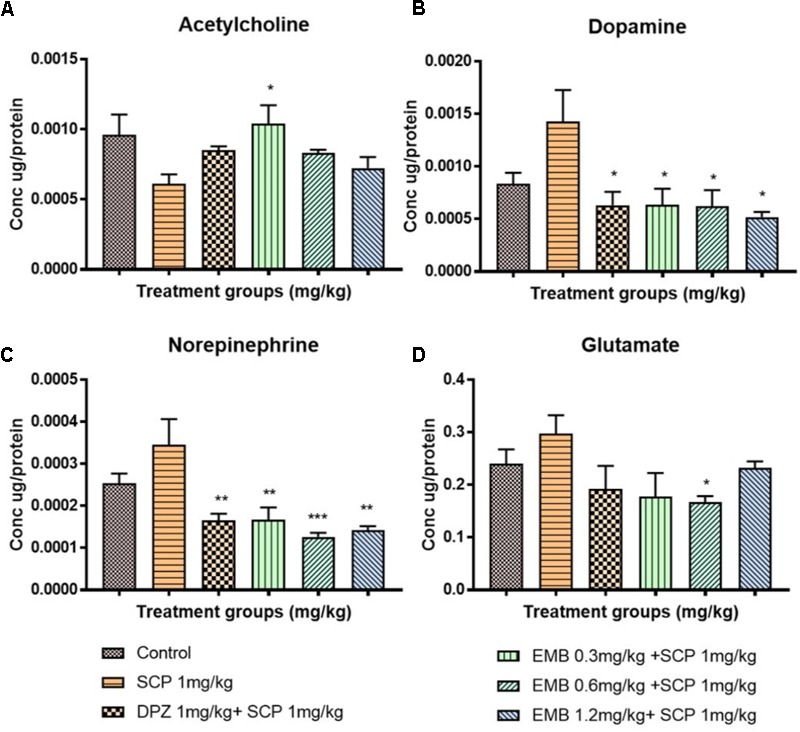
The concentration of neurotransmitters in the rat hippocampi after chronic scopolamine. The figure represents the rat hippocampal neurotransmitter levels of **(A)** Acetylcholine, **(B)** Dopamine, **(C)** Norepinephrine, and **(D)** Glutamate. All changes in the neurotransmitter levels were compared to the negative control group (SCP 1 mg/kg). Data are expressed as Mean ± SEM, *n* = 4 and statistical analysis by one-way ANOVA followed by Dunnett test ^∗^*P* < 0.05, ^∗∗^*P* < 0.01, and ^∗∗∗^*P* < 0.001.

Administration of scopolamine significantly altered the levels of ACh, DA, NE, and Glu in the rat brain’s hippocampus. Specifically, the level of ACh (*P* < 0.05) decreased substantially whereas other neurotransmitters’ levels increased significantly (*P* < 0.05 for DA and Glu; *P* < 0.01 for NE). Nevertheless, embelin treatment significantly normalized the level of all these neurotransmitters, and it was in a dose-dependent manner for ACh and DA (**Figures 5A–D**) (^∗^*P* < 0.05, ^∗∗^*P* < 0.01, and ^∗∗∗^*P* < 0.001).

### Neurogenesis and Lipid Peroxidation in the Hippocampus

**FIGURE 6:**
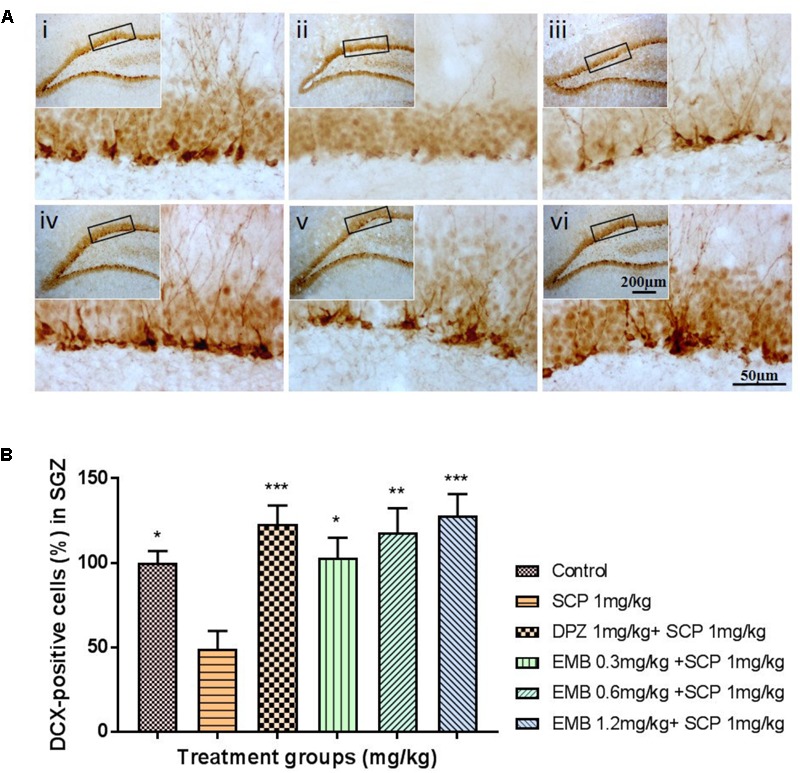
DCX immunohistochemical analysis of the effects of embelin in improving scopolamine-induced suppression of neurogenesis in the dentate gyrus. **(A)** DCX-positive staining in immature neurons is shown in the subgranular zone of the dentate gyrus. Photomicrographs of the hippocampal section of treatment groups was (i) Control (ii) SCP 1 mg/kg alone (iii) DPZ 1 mg/kg + SCP 1 mg/kg (iv) EMB 0.3 mg/kg + SCP 1 mg/kg (v) EMB 0.6 mg/kg + SCP 1 mg/kg (vi) EMB 1.2 mg/kg + SCP 1 mg/kg. Representative photomicrographs were taken at magnifications of 40X and 200X. **(B)** Quantification of DCX population. Data are expressed as means Mean ± SEM, *n* = 5 and statistical analysis by one-way ANOVA followed by Dunnett test ^∗^*P* < 0.05, ^∗∗^*P* < 0.01, and ^∗∗∗^*P* < 0.001.

**FIGURE 7:**
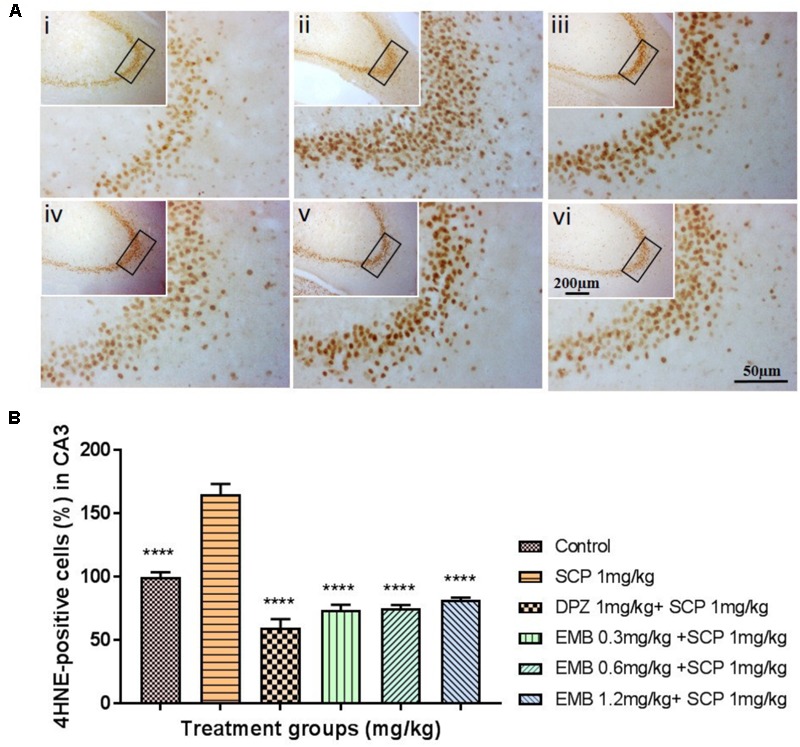
4HNE immunohistochemical analysis illustrating the inhibitory effects of embelin against scopolamine-induced lipid peroxidation in the hippocampus. **(A)** The 4HNE-positive stained cells in the CA3 region of the hippocampus are indicative of lipid peroxidation. Photomicrographs hippocampal section of treatment groups was (i) Control (ii) SCP 1 mg/kg alone (iii) DPZ 1 mg/kg + SCP 1 mg/kg (iv) EMB 0.3 mg/kg + SCP 1 mg/kg (v) EMB 0.6 mg/kg + SCP 1 mg/kg (vi) EMB 1.2 mg/kg + SCP 1 mg/kg. Representative photomicrographs were taken at magnifications of 40X, and 200X. **(B)** Quantification of 4HNE protein in CA3. Data are expressed as means Mean ± SEM, *n* = 5 and statistical analysis by one-way ANOVA followed by Dunnett test ^∗^*P* < 0.05, ^∗∗^*P* < 0.01, ^∗∗∗^*P* < 0.001, and ^∗∗∗∗^*P* < 0.0001.

Scopolamine significantly inhibited adult neurogenesis via a reduction in the distribution of dendrites and neuron bodies in the dentate gyrus (DG) region, as shown by DCX staining in the subgranular zone (SGZ) (**Figure 6A**). Pretreatment with embelin totally ameliorated adult neurogenesis by enhancing immature neurons in the SGZ in a dose-dependent approach in comparison with the negative control (*P* < 0.05 for 0.3 mg/kg, *P* < 0.01 for 0.6 mg/kg and *P* < 0.001 for 1.2 mg/kg; **Figure 6B**). On the other hand, scopolamine injection significantly induced lipid peroxidation in the hippocampus, as represented by a deep brown color in the cornu ammonis 3 (CA3) regions through 4HNE staining. Pretreatment with embelin significantly lowered 4HNE-positive staining in the CA3 (threefold change) compared with the negative group (*P* < 0.0001 for all embelin groups; **Figures 7A,B**). Besides that, donepezil ameliorated these alterations triggered by scopolamine, as displayed through both DCX and 4-HNE staining.

## Discussion

This work aims to determine whether embelin has an anti-amnesic effect by modulating the cholinergic pathway. An animal model of hippocampal memory damage due to intraperitoneal injection of scopolamine was adopted to verify this hypothesis. The experiments comprised of two parts: Experiment 1 (pretreatment with embelin without scopolamine injection during training) to test embelin’s nootropic effects on learning and memory process, and Experiment 2 (multiple exposures of scopolamine injection) to assess the effect of embelin on anti-amnesic activities and biochemical aspects during learning and memory process.

At the beginning of this experiment, we conducted a dose deciding study to find the therapeutic dose of embelin. A prior literature search determined that the range of embelin dose was between 2.5 mg/kg to 10 mg/kg for the intraperitoneal route in CNS related animal models (Mahendran et al., 2011; Afzal et al., 2012). However, our preliminary study using these range of embelin doses resulted in a neurobehavioral effect on coordination and motor activity whereby the treated rats were immobile and kept falling from the behavioral apparatus. Thus, we decided 1.2 mg/kg as the highest dose as the LD_50_ value for embelin was 44 mg/kg for intraperitoneal administration reported by Poojari (2014). Furthermore, we decided 0.3 mg/kg and 0.6 mg/kg would be the low dose and medium dose respectively, and all these 3 doses were effective therapeutic doses for our study as we noticed no side effects.

In this experiment, NOR and EPM were applied as behavioral models to evaluate learning and memory. The NOR test is particularly relevant in AD research as it allows the assessment of visual recognition memory, which is affected early in AD progression, involving brain regions similar to those affected by this devastating and debilitating neurodegenerative disease (Grayson et al., 2015). On the other hand, EPM is a behavioral test employed to study long-term spatial memory (Uddin et al., 2016b). Certain EPM parameters like retention transfer latency are utilized for the evaluation of memory. A decrease in transfer latency on the second day, which is after 24 h, indicates an improvement of memory and vice-versa (Dhingra and Kumar, 2012). The findings of this study showed that embelin at 0.6 mg/kg displayed nootropic activity in both the recognition index and inflection ratio in the NOR and EPM tests, respectively (**Figures 2A,B**). However, the nootropic activity of embelin in both behavioral paradigms was found to be dose independent. This could be explained that at a higher dose, the drug reaches its maximum effect so increasing the drug dosage does not increase its effectiveness, but on the contrary, effectiveness decreases. This theory is supported by the fact that CNS drugs such as antipsychotic drugs produce maximum dopaminergic blockage at high doses. However, further dose increments will not produce any dopamine blockage but eventually lead to other side effects such as anticholinergic activity (Bridges, 1981). It is possible that in this experiment, the 1.2 mg/kg embelin group has reached its maximum effect and therefore cognitive ability has declined. Based on the behavioral results obtained, it can be suggested that embelin is a nootropic drug that acts as a natural cognitive enhancer. These findings show that supplementation of embelin significantly amplified the rats’ memory function and 0.6 mg/kg of embelin demonstrated significant nootropics effects. Nootropic drugs are used to treat cognition deficits in patients with AD, schizophrenia, stroke, attention deficit hyperactivity disorder (ADHD), and vascular dementia (VaD) (Birks and Grimley Evans, 2009; Froestl et al., 2012).

Scopolamine-induced dementia has been used extensively to assess potential therapeutic agents for treating AD (Kwon et al., 2009). Scopolamine is a nonselective muscarinic cholinergic receptor antagonist associated with cholinergic dysfunction, which causes performance deficits in learning and memory (Heo et al., 2014). Therefore, in this study, scopolamine was administered to rodents for 1 week to induce cholinergic neurodegeneration along with cognitive deficits. Following 6 days of scopolamine administration, the scopolamine treated group had less than 20% of the recognition index of other groups. Pretreatment with embelin ameliorated memory impairment caused by scopolamine (**Figure 3A**), with the recognition index being twofold more, in comparison with scopolamine treated group in a dose-dependent manner. These results exposed that embelin was as effective as the donepezil-treated group. Moreover, the findings showed that embelin treatment attenuated amnesic behavior in EPM, but it was insignificant (**Figure 3B**). Hence, these outcomes suggest that embelin had an anti-amnesic effect in the scopolamine model.

The brain is susceptible to oxidative stress because it consumes huge amounts of oxygen, has an abundant lipid content, and a low antioxidant level compared than other organs (Serrano and Klann, 2004). Furthermore, it is well known that the hippocampus region in the brain is crucial for learning and memory, and the formation of spatial memory (Huang et al., 2015; Lee et al., 2016). The scopolamine-induced memory deficit model demonstrated that prominent oxidative stress and memory deficits in a rodent model is similar to that in AD patients, even though the mechanism of action remains unclear (Lee et al., 2015). The change in the mRNA levels of antioxidants in the hippocampus after embelin pretreatment was examined using the scopolamine model in this present study. Scopolamine injection induced oxidative stress in the hippocampus, as evident by the decreased levels of CAT and SOD1 mRNA levels in the scopolamine alone treated negative group. To prevent or slow down the progression of free radical-mediated oxidative stress, brain antioxidant defense enzymes such as CAT and SOD play a vital role in protecting tissues against oxidative damage (Uddin et al., 2016a). Antioxidant mRNA alteration caused by scopolamine injection was significantly ameliorated for SOD1 via pretreatment with embelin. However, CAT mRNA level was decreased by scopolamine induction, but it was not significant (**Figures 4C,D**). Additionally, scopolamine-induced lipid peroxidation in the hippocampus’s CA3 was shown as positively stained 4HNE cells. Nonetheless, pretreatment with embelin completely attenuated the over-production of 4HNE cells (**Figures 7A,B**). These results propose that the protective antioxidant gene response by embelin pretreatment reduced lipid peroxidation induced by scopolamine. An increase in 4HNE cells is a key histopathological feature of neurodegenerative diseases like AD (Serrano and Klann, 2004).

Expression of BDNF and CREB1 mRNA levels in scopolamine-induced hippocampal tissue were examined to investigate the role of embelin in neurogenesis and synaptic plasticity. In this study, hippocampal BDNF and CREB1 were markedly reduced due to scopolamine injection, and pretreatment with embelin increased the mRNA expression level of both BDNF and CREB1. A high dose of embelin at 1.2 mg/kg exhibited maximum protection by increasing the levels of BDNF and CREB1. Other than that, cAMP response element binding protein (CREB) plays a crucial role in neuronal growth, proliferation, differentiation, and survival (Lee et al., 2016). In our results, the explanation for the increased in dose dependency for both BDNF and CREB 1 could possibly be that embelin may be responsible for visual recognition memory in NOR through this BDNF/CREB pathway. We noticed that at the 1.2 mg/kg dose, embelin expressed high mRNA levels of BDNF and CREB1 and this could be the reason for a 60% increase in visual recognition index in NOR when compared with the scopolamine treated group. Thus, this validates the role of BDNF-CREB signaling in visual recognition memory, particularly for hippocampus-dependent learning.

Likewise, adult hippocampal neurogenesis plays a key role in hippocampal memory function (Mu and Gage, 2011). Altman and Das (1965) first reported on the continual production of new neurons in the adult hippocampus. These new neurons originated from adult neural stem cells (NSCs) residing in the SGZ of DG (Bonaguidi et al., 2011). In this present research, a significantly reduced level of immature neurons, revealed through DCX staining of scopolamine-induced rat hippocampus was determined, while pretreatment with embelin distinctly ameliorated repression of the SGZ region’s neuronal precursor cells in a dose-dependent manner (**Figures 6A,B**).

Numerous studies have reported that most classical neurotransmitter systems such as ACh, NE, Glu, and DA, influence learning and memory (Myhrer, 2003). We adopted LC-MS/MS method as it is a simple, sensitive and simultaneously able to quantify the four major neurotransmitters from rat hippocampal tissue in a single run (Zheng et al., 2012). The extraction of the neurotransmitters from rat hippocampus was done with utmost care and prior to LC-MS/MS analysis to avoid any possibilities of sample degradation and oxidation as described by He et al. (2013). The neurotransmitters’ concentrations were expressed as a ratio of total protein concentration in order to get correct value and to avoid possible variation in sample when subjected to LC-MS/MS. In AD patients, pathological changes affecting glutamatergic, cholinergic, noradrenergic, and serotonergic systems have been revealed (Francis et al., 1999). In this study, the effect of embelin on brain neurotransmitter levels in rats administered scopolamine was investigated. ACh plays an essential role in learning process and memory as a key transmitter in the cholinergic system (Chen et al., 2016). A decrease in ACh levels is reported in this study as a biomarker of scopolamine-induced cognitive impairment in the rat hippocampus. Embelin administered at a dose of 0.3 mg/kg significantly increased ACh levels, subsequently improving cholinergic function. Interestingly, Arora and Deshmukh (2017) reported that embelin treatment in a streptozotocin-induced rat model decreased AChE activity, which is the enzyme that metabolizes ACh into choline and acetate. Therefore, a reduction in AChE level indicates a high level of ACh as a result of embelin treatment, which is similar to our results. In the current research, Glu levels were raised after being treated with scopolamine. Similar outcomes were reported by Pandareesh et al. (2016) and Arora and Deshmukh (2017). Administration of 0.6 mg/kg embelin significantly lowered the level of Glu. A rise in Glu level has been reported to cause excitotoxic neuronal damage and loss of cognitive function (Arora and Deshmukh, 2017) and also associated with excitotoxicity in AD brains (Jackson, 2014). Scopolamine treatment also caused an increment in the levels of DA and NE in the hippocampus. Earlier reports suggests that an increase in DA and NE levels leads to amnesia and memory deficits. Wu et al.’s (2014) study, demonstrated that donepezil treatment can modulate the increase levels of DA and NE in disease control group. Interestingly, a similar protective effect was observed with embelin pre-treatment in amnesia condition.

In this scopolamine model, our results are unusual, with embelin causing different dose dependency in the behavioral model and neurotransmitters, particularly ACh when compared to other reported studies that utilized embelin. This could be explained by embelin being neuroprotective in a scopolamine-induced amnesia model via visual recognition memory but not in long-term spatial memory. This theory is supported by our results as there was a dose dependency in embelin treatment in NOR and the result of embelin is comparable with the donepezil group. However, we could not see this pattern in EPM. Whilst embelin improved visual recognition in dose dependency manner, it also reduced the level of ACh in a dose-dependent manner as well. This discrepancy could be because at a dose of 0.3 mg/kg, embelin might be effectively increasing the level of ACh but stops further production of ACh at 1.2 mg/kg. At this particular dose, embelin probably plays a different role in inhibiting the enzyme AChE. This could be the reason that at 1.2 mg/kg of embelin, we observed a high recognition index in NOR of scopolamine-induced amnesia rats.

## Conclusion

**FIGURE 8:**
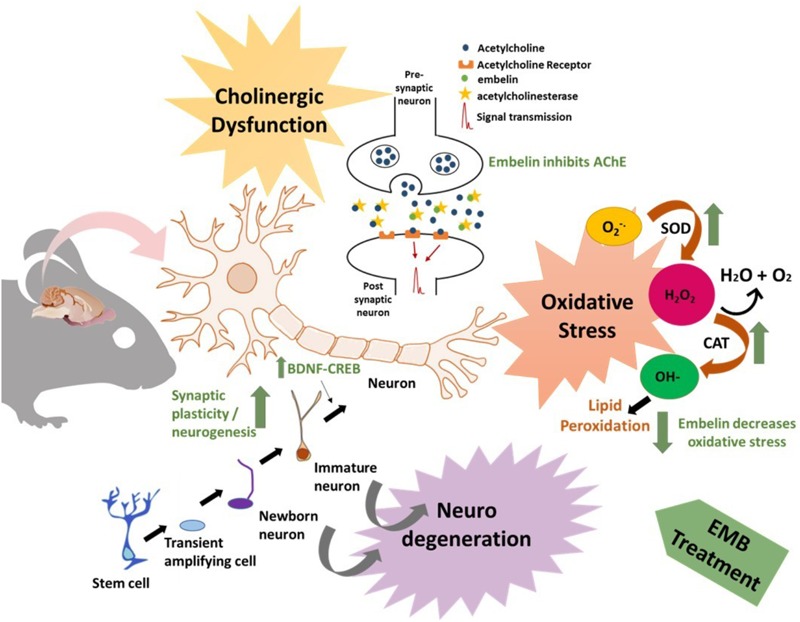
Schematic diagram showing the effect of embelin in a diseased condition. The figure depicts cholinergic dysfunction, oxidative stress, and neurodegeneration which contributes to neuronal loss, synaptic dysfunction, and an AD-like condition. Embelin could act as memory enhancer by stimulating the cholinergic systems via acetylcholine release and inhibition of AChE. In the oxidative defense pathway, embelin reduces oxidative stress by increasing SOD and CAT mRNA levels and eventually reducing lipid peroxidation. In addition to these effects, a rise in DCX expression by embelin treatment contributes to neurogenesis and an increase in BDNF-CREB levels may contribute to synaptic plasticity.

In conclusion, the results from this study have demonstrated that embelin displays nootropic and neuroprotective abilities in scopolamine-induced amnesia in rats. Nootropic effects may be attributed to an increase in visual recognition and spatial memory in both NOR and EPM. Embelin possesses anti-amnesic effects, which could be mediated by an antioxidant gene response particularly though SOD1, the CREB-BDNF pathway, hippocampal neurogenesis, and cholinergic activity. The anti-amnesic effect of embelin is also comparable to that of donepezil at a specific concentration even though it is not in a dose-dependent manner in certain cases. Therefore, embelin could be a promising treatment for patients suffering from neurodegenerative diseases. **Figure 8** shows the potential mechanism of action of embelin in scopolamine-induced memory impairment in rodents.

## Ethics Statement

The experimental protocol was approved by the Monash Animal Research Platform (MARP) Animal Ethics Committee, Monash University, Australia (MARP/2016/054).

## Author Contributions

SB performed all the experiments and was responsible for the writing of the manuscript in its entirety. YK helped in designing gene expression study, result analysis and figures in the manuscript. IO helped in LC-MS/MS method. MS helped in conceptualizing, designing the study, result analysis, and manuscript writing. All authors gave their final approval for the submission of the manuscript.

## Conflict of Interest Statement

The authors declare that the research was conducted in the absence of any commercial or financial relationships that could be construed as a potential conflict of interest.
